# Endoscopically Guided Laparoscopic Gastrojejunostomy Tube Placement for Patients with Distal Esophageal Stents

**DOI:** 10.1007/s11605-017-3379-0

**Published:** 2017-02-08

**Authors:** Marlieke E. Nussenbaum, Edward Y. Chan, Min P. Kim, Puja G. Khaitan

**Affiliations:** 1 Division of Thoracic Surgery, Department of Surgery, Houston Methodist Hospital, 6550 Fannin Street, Suite 1661, Houston, TX 77030 USA; 2 Weill Cornell Medicine, Houston Methodist Hospital, Houston, TX USA

**Keywords:** Esophageal perforation, Esophageal stents, Feeding access (gastrojejunostomy tube placement)

## Abstract

Patients with distal esophageal pathology such as perforation, trachea-esophageal fistulae, and/ or obstructing gastroesophageal junction tumor present a challenging situation in terms of feeding access where an esophageal stent is placed across the gastroesophageal junction. In order to allow simultaneous gastric decompression and post-pyloric feeds without significant reflux up through the stent, a gastrojejunostomy (GJ) tube is a viable option. We hereby describe a hybrid approach to placing these GJ tubes in this cohort of patients using simultaneous laparoscopy, endoscopy, and fluoroscopy with minimal manipulation of the stent itself. We have employed this technique of placing GJ tubes 2–3 days following placement of the esophageal stent in six consecutive patients. All patients tolerated the procedure well without any complications. Endoscopically guided laparoscopic GJ tubes are ideal for bridging patients, with distal esophageal pathology requiring esophageal stents, to oral intake.

**Electronic supplementary material:**

The online version of this article (doi:10.1007/s11605-017-3379-0) contains supplementary material, which is available to authorized users.

## Introduction

The most common long-term route of enteral feeds is via a gastrostomy or a jejunostomy tube (G- or J-tube). However, there are clinical situations where a patient requires gastric decompression via a nasogastric tube or G-tube with simultaneous distal feeds. While nasoduodenal or nasojejunal tubes can be safely placed for post-pyloric feeds, they are notorious for getting displaced and/or clogged due to their small caliber,^1^^,^ 2 making discharging a patient home difficult. Additionally, while a J-tube could adequately provide distal enteral feeds, one would still require either a nasogastric tube or G-tube to vent the gastric cavity. In such cases, the nasogastric tube either crosses an indwelling stent which is not ideal or patients have two enteral accesses (G-tube to vent and J-tube for feeds since gastric feeds are not acceptable given risk of reflux through the esophageal stent). In such situations, a gastrojejunostomy (GJ) feeding tube is an ideal feeding access.

Historically, GJ feeding tubes have been placed at our institution in two phases for this select group of patients. First, a G-tube had to be placed endoscopically that potentially risked displacement of the esophageal stent and once the track matured after 10–14 days, interventional radiologists threaded a post-pyloric jejunal feeding tube under fluoroscopy through the G-tube or swapped it out for a GJ tube. However, this resulted in a delay in enteral nutrition, long-term dependence on parenteral nutrition with risk of line infection, and associated cholestasis, as well as prolonged hospital stay while the patient waited to have the jejunal extension placed.

Here, we present our hybrid technique where a GJ feeding tube is placed laparoscopically with simultaneous use of an endoscope. Since G-tubes are anecdotally better tolerated by patients than a J-tube in terms of tube dislodgment and obstruction,^3^^,^ 4 GJ tubes are an excellent alternative since they more or less behave as G tubes.

## Technique

**Fig. 1:**
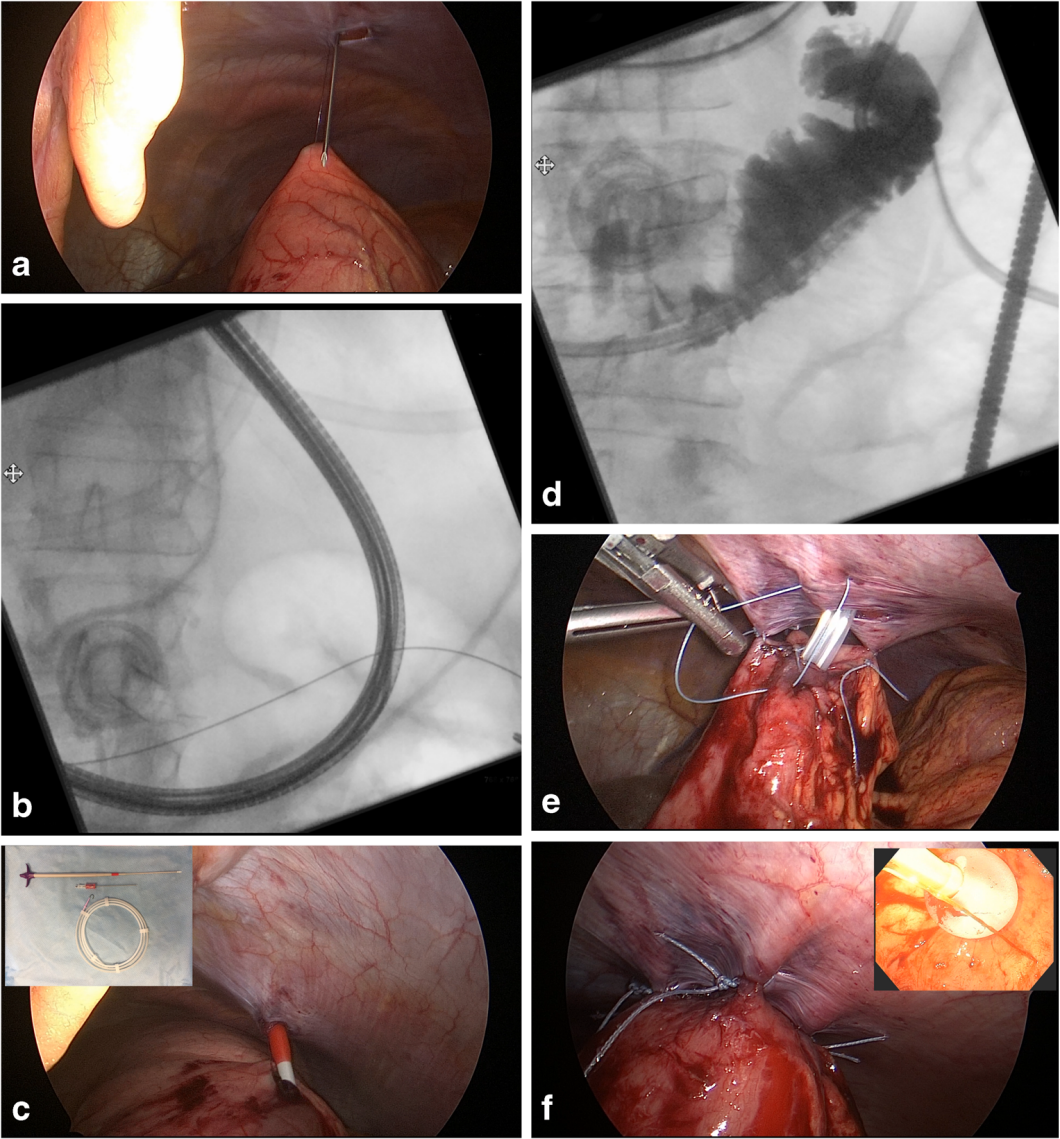
Using a T-fastener, the stomach is stabilized while a large-bore needle is used to pass a guidewire into the gastric lumen (**a**). This wire is then guided post-pyloric into the proximal small bowel and confirmed to be in appropriate position with on-table fluoroscopy (**b**). Using a 22Fr peel-away dilator sheath (**c**), an 18Fr GJ tube is threaded into the stomach over the wire. The final position of the GJ tube is confirmed to be in place by injecting contrast into the J-port (**d**). The anterior wall of the stomach is then anchored to the abdominal wall (**e**), and the gastric balloon is inflated (**f**)

The procedure begins laparoscopically where one 12-mm peri-umbilical port is placed in the midline and two 5-mm ports are placed in the right upper quadrant and right lower quadrant. Endoscopy is performed once pneumoperitoneum is in place and a GJ tube insertion site is identified in the body of the stomach along the greater curvature. Using a stitch or T-fastener (Kimberly-Clark, Roswell, GA; surgeon preference), the stomach is stabilized while a large-bore needle is used to pass a guidewire into the gastric lumen (Fig. 1a). This wire is then grasped with an endoscopic forceps and guided post-pyloric into the proximal small bowel. The distal end of the wire is confirmed to be in the small bowel with on-table fluoroscopy (Fig. 1b). Next, an 18Fr GJ tube (Kimberly-Clark, Roswell, GA) is inserted using Seldinger technique with a 22Fr peel-away dilator sheath kit (Kimberly-Clark, Roswell, GA; Fig. 1c). The J-limb of the GJ tube is passed distally over the wire and confirmed to be in place using fluoroscopy (Fig. 1d). The anterior wall of the stomach is finally anchored to the abdominal wall in a Stamm gastrostomy fashion (Fig. 1e) and the gastric balloon is inflated against the abdominal wall (Fig. 1f). The gastric port is immediately placed on bedside gravity and the jejunal port can be used for enteral feeds the next day.

### Patients

Patient selection is key. Most of the patients selected for this route of feeding had distal esophageal pathology who required esophageal stents precluding them from eating or getting gastric feeds that can reflux. Jejunal enteral feeds were a superior mode of nutrition for this cohort of patients, when compared to long-term parenteral route.

### Results

As seen in Table 1, we performed this procedure consecutively on six patients over the last 6 months. All of them were either those who suffered from distal esophageal perforation and had esophageal stents placed crossing the gastroesophageal junction (GEJ, *n* = 3) or those with advanced esophageal or lung cancer with tracheoesophageal fistula requiring an esophageal stent (*n* = 2). One patient had developed an obstructing esophageal cancer at the GEJ 4 years after her bilateral lung transplant, who needed a palliative stent during definitive chemoradiation. All of them did well and only the three patients who suffered from spontaneous/iatrogenic distal esophageal perforation were able to get the GJ tube removed once their stents were removed and they were tolerating oral intake without any problems.

**Table 1:** Our experience: outcomes of patients undergoing gastrojejunostomy feeding tube placement

Age/Sex	Indication	Stent placed?	GJ tube removed?
74/F	Boerhaave syndrome; left thoracotomy, chest washout	Yes	Yes
41/M	Boerhaave syndrome; left thoracoscopic mediastinal washout	Yes	Yes
58/M	Stage IIIA lung cancer s/p adjuvant radiation only with recurrence in subcarinal region, then with chemoradiation, now with TEF and PEG tube	Yes	No
76/M	Stage IIIA lung cancer with subcarinal involvement with subsequent BPF and TEF, no neoadjuvant or adjuvant chemoradiation	Yes	No
59/F	Newly diagnosed esophageal cancer and stricture, history of bilateral lung transplant 4 years ago	Yes	No
82/F	Iatrogenic esophageal perforation from TEE probe (during MVP)	Yes	Yes

*Abbreviations: BPF* bronchopleural fistula, *MVP* mitral valvuloplasty, *PEG* percutaneous gastrostomy, *TEE* trachea-esophageal echocardiogram probe, *TEF* tracheoesophageal fistula

After a median follow-up period of 8 months, of the six reported patients, none of them had a complication from the GJ tube itself, in terms of migration, occlusion, reflux, or dislodgement of the tube. Albeit, this is a very commonly noted problem of any J tube extension or J-limb of the GJ tube, which would require replacement of the GJ tube by interventional radiology. One patient succumbed to death given that she was end-stage esophageal cancer, status post bilateral lung transplant, and on immunosuppression; and the stent and feeding tube had been placed as palliation efforts.

## Discussion

There are several notable advantages of this procedure. Firstly, both gastric decompression and post-pyloric feeds can be accomplished with one tube. Secondly, the tube is placed safely and under direct visualization in one stage. Lastly, given the large caliber of the tube, clogging of the tube is less of an issue and because it is not a tube-within-a-tube, no manipulation of the tube is required in the immediate post-placement period. These tubes can be easily exchanged over a wire by interventional radiology, if needed in case of clogged or dislodged tube. On the contrary, the procedure does require two competent operators to simultaneously perform the endoscopic driving of the wire into the small bowel while placing the GJ tube laparoscopically.

While this procedure is safe, patient selection is important. Patients who are not good candidates for general anesthesia, laparoscopy, or have difficult foregut anatomy should not undergo GJ tube placement and maybe better off with parenteral nutrition. Endoscopically guided laparoscopic GJ tubes are a viable option for patients who require both gastric decompression and post-pyloric feeds, and are ideal for bridging patients to oral intake.

## Electronic supplementary material

Below is the link to the electronic supplementary material.

Video 1 Step-by-step technique for placement of endoscopically-guided laparoscopic gastrojejunostomy tube placement. (MOV 294688kb)
